# Low Self-Confidence and Diabetes Mismanagement in Youth with Type 1 Diabetes Mediate the Relationship between Behavioral Problems and Elevated HbA1c

**DOI:** 10.1155/2016/3159103

**Published:** 2015-11-23

**Authors:** Minke M. A. Eilander, Maartje de Wit, Joost Rotteveel, Frank J. Snoek

**Affiliations:** ^1^Department of Medical Psychology, VU University Medical Center, De Boelelaan 1117, 1081 HV Amsterdam, Netherlands; ^2^EMGO+ Institute for Health and Care Research, VU University Medical Center, Van der Boechorststraat 7, 1081 BT Amsterdam, Netherlands; ^3^Department of Pediatrics, VU Medical Center, De Boelelaan 1118, 1081 HV Amsterdam, Netherlands; ^4^Department of Medical Psychology, Academic Medical Center (AMC), Meibergdreef 9, 1100 DD Amsterdam, Netherlands

## Abstract

*Introduction*. Previous studies indicated an association between behavior problems (internalizing, externalizing) and glycemic control (HbA1c) in youth with type 1 diabetes (T1D). The aim of this study is to examine if this association is mediated by self-confidence and mismanagement of diabetes. *Methods*. Problem behavior was assessed with the Strengths and Difficulties Questionnaire. Mediating variables were assessed using the Confidence in Diabetes Self-Care-Youth and Diabetes Mismanagement Questionnaire. HbA1c was derived from hospital charts. Bootstrap mediation analysis for multiple mediation was utilized. *Results*. 88 youths with T1D, age 11–15 y, participated. The relation between both overall problem behavior and externalizing behavior problems and HbA1c was mediated through *confidence in diabetes self-care* and *mismanagement* (*a*_1_ *b*_1_ + *a*_2_ *b*_2_ *path*; point estimate = 0.50 BCa CI 95% 0.25–0.85; *a*_1_ *b*_1_ + *a*_2_ *b*_2_ *path*; point estimate = 0.73 BCa CI 95% 0.36–1.25). *Conclusions*. Increased problem behavior in youth with T1D is associated with elevated HbA1c and mediated by low self-confidence and diabetes mismanagement. Screening for problem behavior and mismanagement and assisting young patients in building confidence seem indicated to optimize glycemic control.

## 1. Introduction

Adolescence is characterized by major biological and psychosocial changes, which interact in complex ways. This is particularly true for youth with type 1 diabetes (T1D) and probably explains the fact that youth with diabetes have the poorest glycemic control of all age groups, with less than 15% of adolescents with type T1D reaching Hemoglobin A1c (HbA1c) levels below target [1–5]. On top of the hormonal changes that negatively affect blood glucose control, adolescents with T1D have an increased risk of developing depression, anxiety, and disturbed eating behaviors, relative to healthy youth. These problems typically occur in mid-adolescence [6] and result in poor glycemic control [7–11]. Externalizing behavior problems (e.g., attention and disruptive behavior complaints) are associated with decreased glycemic control as well [12–15]. Although behavior problems at diagnosis do not seem to impact lifelong poor glycemic control [16], they have been found to be directly associated with hyperglycemia [15]. Adolescents showing external problem behavior seem to be generally unresponsive to punishment, are often impulsive, and have concentration problems [17]. Problematic behavior, both internal and external, frequently coincides with low self-efficacy beliefs, low self-esteem, dysfunctional coping mechanisms, and increased risk taking behavior, all complicating daily self-management of diabetes [17]. Self-efficacy beliefs, for example, low self-confidence, and diabetes mismanagement are likely to mediate the relationship between behavior problems and poor glycemic outcomes, but this hypothesis has not been previously tested.

Using baseline data of multicenter cohort DINO study (diabetes in development) that examines the complex interaction between biological and psychosocial development during adolescence [18], we examined whether overall, external, and internal problem behavior are associated with glycemic control and whether this relationship is mediated by confidence in diabetes self-care and mismanagement.

## 2. Materials and Methods

### 2.1. Participants

Youth aged 8 to 15 treated (*N* = 598) in 5 pediatric diabetes centers in Netherlands were invited to participate in the DINO study. The participating centers provide secondary and tertiary clinical care to children and adolescents with T1D in their region and can be considered representative of youth with T1D in Netherlands. Exclusion criteria were mental retardation, diabetes other than type 1, and diagnosis less than 6 months prior to the start of the study. Written informed consent was obtained from all parents and adolescents 12 years and older. Participants completed an online survey. In view of their age, 8–11-year-olds completed a shorter survey than participants 11 years and older. Data from the latter survey were used for the study reported in this paper. In total, 151 children and adolescents (25.3%) agreed to participate, of whom 100 were ≥11 years.

The DINO study was approved by the Medical Ethical Committee of VU University Medical Center.

### 2.2. Measures

*Problem behavior* was assessed using the Strengths and Difficulties Questionnaire (SDQ). SDQ [19, 20] captures emotional and behavioral functioning and contains 25 items, rated on a 3-point Likert scale (e.g., “Other people my age generally like me”). The SDQ comprises five scales: emotional symptoms, conduct problems, hyperactivity/inattention, peer relationship problems, and prosocial behavior. The overall score of problem behavior (range 0–40) can be divided into external (range 0–20) and internal (range 0–20) problem behavior. Cronbach's *α* was 0.70 on the overall scale [19, 20], in the current study 0.60. Higher scores indicate more problematic behavior; scores ≤ 13 are considered normal.

*Confidence in diabetes self-care* was assessed using an adapted adolescent version of the Confidence in Diabetes Self-Care Scale (CIDS) [21]. The original adult version of the CIDS consists of 20 items on a 5-point Likert scale, Cronbach's *α* = 0.86 [21]. The adapted youth version consists of 12 items (e.g., “I believe I can check my blood glucose at least 2 times a day”): 10 of the original questionnaire, 2 items combined to 1 (original questions 2 and 20), and 1 additional item regarding alternations in blood glucose. Cronbach's *α* of the CIDS-Youth in the current study was 0.79. Higher scores represent higher diabetes self-confidence (range 12–60).

*Mismanagement in diabetes self-care* was assessed using an adapted version of the mismanagement scale [22]. The original version consists of 10 items of which 3 items were used and 1 was adjusted. The recall period was changed from 10 days to the past week. Answers are given on a 5-point Likert scale (e.g., “In the past 7 days, how often did you miss shots/did not bolus?”). Cronbach's *α* of the original version is 0.74 and 0.60, respectively [22]. Cronbach's *α* of the adapted version in the current study was 0.47. Higher scores indicate more mismanagement (range 4–16).

*Demographic and diabetes related data *were derived from hospital charts during the same period as the completion of the survey. HbA1c was used as a marker of* glycemic control* over the past 8–12 weeks, with recommended target < 7.5%, 58 mmol/mol [23].

### 2.3. Analyses

*t*-tests and chi-square tests were applied in order to examine differences in HbA1c, age, and gender between responding and nonresponding adolescents. To examine whether there was a relationship between problem behavior (overall, external, and internal) and glycemic control and whether this relationship is mediated by confidence in diabetes self-care and diabetes mismanagement, bootstrap mediation analysis for multiple mediation through the Indirect Macro was applied [24, 25], correcting for age and gender. Since we chose to use more than one possible mediator, this method was considered more appropriate than traditional models [24, 26–28]. Analyses were performed with a bootstrap of 5000 resamples, in which random samples based on the original data are generated. A 95% confidence interval (CI) was calculated [25].

## 3. Results

A total of 88 adolescents (45 boys) completed the online survey (88.0% of the 100 youths ≥ 11 y), mean age 12.9 (±1.2) years with a mean disease duration of almost 6 years. Baseline characteristics are shown in Table 1. There were no differences in HbA1c, age, and gender between responders and nonresponders. Thirteen adolescents (14.8%) reported problem behavior above the normal range (overall problem behavior score > 13). Almost three-quarters of adolescents (72.7%) had HbA1c levels above target.

### 3.1. Overall Problem Behavior

**Figure 1:**
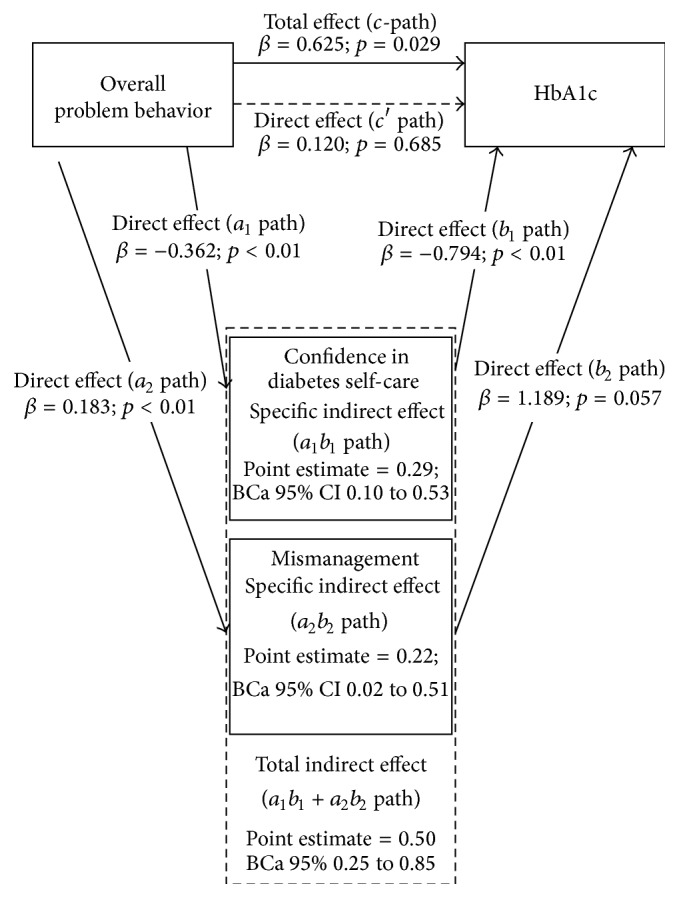
Graphic representation of the multiple mediation model of the associations between overall problem behavior and glycemic control with confidence in diabetes self-care and mismanagement of diabetes self-care. 5000 resamples were calculated while using the bootstrap method [24].

Figure 1 shows the multiple mediation model of the associations between* overall problem behavior* and glycemic control. A significant total effect (*c*-path) was found between overall problem behavior and glycemic control (*β* = 0.625, *p* = 0.029), indicating that higher overall problem behavior scores are associated with higher HbA1c. Mediation analysis showed that this relationship was mediated by confidence in diabetes self-care and mismanagement as the indirect effect was significant (*a*_1_ *b*_1_ + *a*_2_ *b*_2_ *path *point estimate = 0.50, BCa 95% 0.25 to 0.85) and the direct effect (*c*′ path) was not anymore (*β* = 0.120, *p* = 0.685). Increased overall problem behavior was associated with higher confidence in diabetes self-care (*a*_1_ path *β* = −0.362, *p* < 0.01) and worse self-care of diabetes (*a*_2_ path *β* = 0.183, *p* < 0.01). Lower confidence in diabetes self-care was associated with higher HbA1c (*b*_1_ path *β* = −0.794, *p* < 0.01). The association between mismanagement of diabetes and higher HbA1c was borderline significant (*b*_2_ path *β* = 1.189, *p* = 0.057).

### 3.2. External Problem Behavior

**Figure 2:**
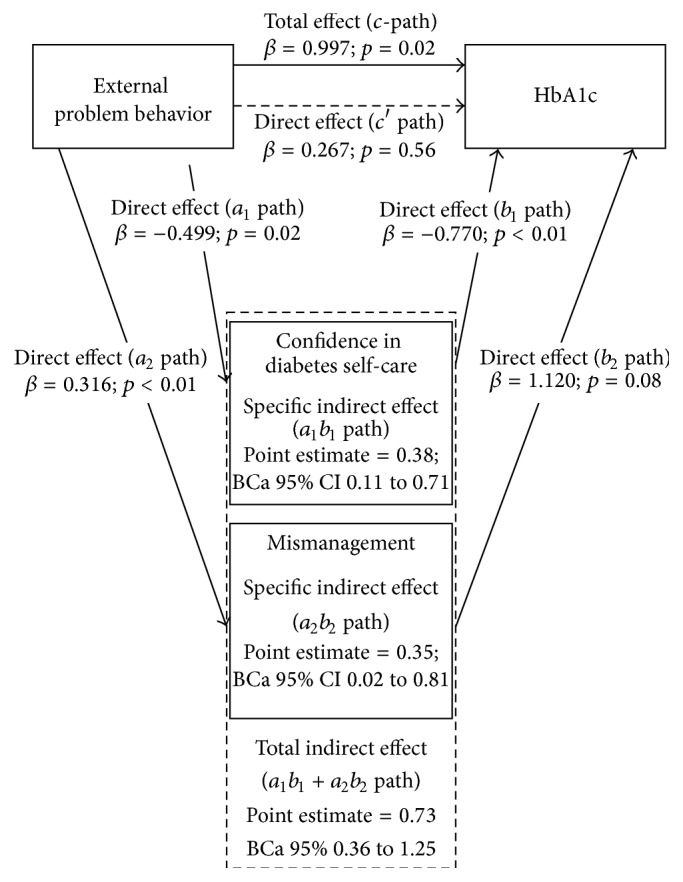
Graphic representation of the multiple mediation model of the associations between external problem behavior and glycemic control. 5000 resamples were calculated while using the bootstrap method [24].

Figure 2 shows the multiple mediation model of the associations between* external problem behavior* and glycemic control. A significant total effect (*c*-path) was found between external problem behavior and glycemic control (*β* = 1.00, *p* = 0.02): increased external problem behavior was associated with higher HbA1c. Again, multiple mediation analysis showed that this relationship was mediated by confidence in diabetes self-care and mismanagement as the indirect effect (*a*_1_ *b*_1_ + *a*_2_ *b*_2_ *path *point estimate = 0.73, BCa 95% 0.36 to 1.25) was significant and the direct effect (*c*′ path *β* = 0.27, *p* = 0.56) was not anymore. Increased external problem behavior was associated with low confidence in diabetes self-care (*a*_1_ path *β* = −0.49, *p* = 0.02) and worse self-management of diabetes (*a*_2_ path *β* = 0.32, *p* < 0.01). Low confidence in diabetes self-care and worse self-management of diabetes were both associated with higher HbA1c; however the latter was not significant (*b*_1_ path *β* = −0.77, *p* < 0.01; *b*_2_ path *β* = 1.12, *p* = 0.08).

### 3.3. Internal Problem Behavior

**Figure 3:**
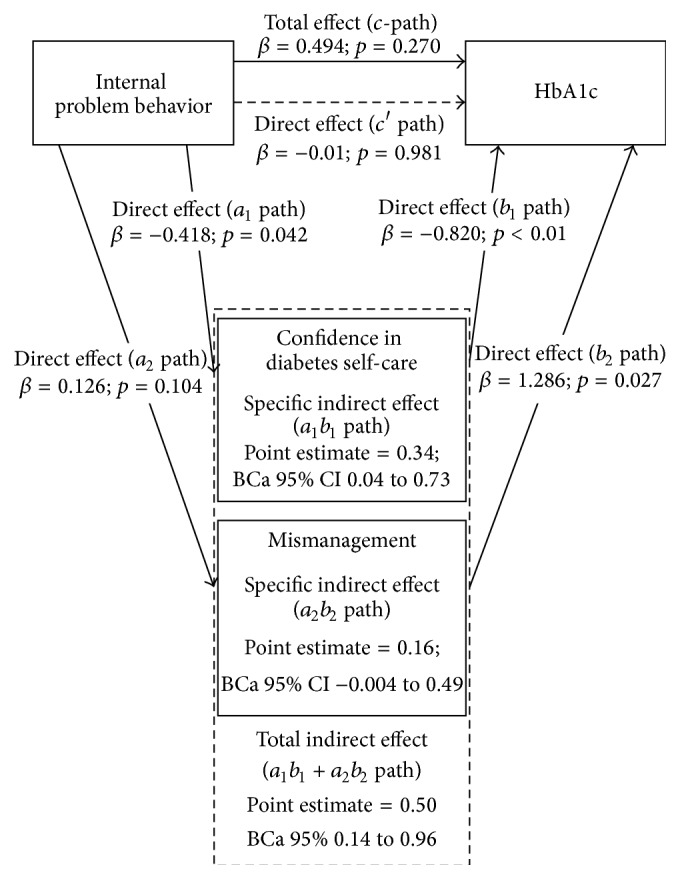
Graphic representation of the multiple mediation model of the associations between internal problem behavior and glycemic control. 5000 resamples were calculated while using the bootstrap method [24].

Figure 3 shows the multiple mediation model of the associations between* internal problem behavior* and glycemic control. In contrast to the overall and external problem behavior, the total effect between internal problem behavior and glycemic control was not significant (*c*-path *β* = 0.494, *p* = 0.270). However, multiple mediation analysis did show a significant mediation by confidence in diabetes self-care and mismanagement as the indirect effect was significant (*a*_1_ *b*_1_ + *a*_2_ *b*_2_ *path *point estimate = 0.50, BCa 95% 0.14 to 0.96). The association of the direct effect (*c*′ path *β* = −0.01, *p* = 0.981) decreased as a result of this mediation. Increased internal problem behavior was associated with low confidence in diabetes self-care (*a*_1_ path *β* = −0.418, *p* = 0.042), but not with worse self-management of diabetes (*a*_2_ path *β* = 0.126, *p* = 0.104). Low confidence in diabetes self-care and worse self-management were both associated with a higher HbA1c (*b*_1_ path *β* = −0.820, *p* < 0.01; *b*_2_ path *β* = 1.286, *p* = 0.027).

## 4. Discussion

The aim of the present study in adolescents with T1D was to investigate whether there is a relationship between problem behavior and glycemic control and whether this relationship is mediated by low confidence in diabetes self-care and mismanagement of diabetes. Increased* overall* and* external* problem behavior were found to be associated with increased HbA1c and these relationships were mediated by confidence in diabetes self-care and self-management of diabetes. Interestingly no total effect was found between* internal* problem behavior and glycemic control, and the relationship between internal problem behavior and diabetes management was not significant; however, the indirect effect was significant. We should interpret these findings with caution, as the relationship could be dose-dependent: the risk of worsened illness management increases when internal problems get more serious [17]. The adolescents participating in our study reported somewhat less problematic behavior on all three SDQ scales (overall, external, and internal problem behavior) compared to the 11–16-year-old adolescents participating in SDQ validation study published in 2003 (overall *M* = 8.6 compared to 9.9, external *M* = 4.9 compared to 5.8, and internal *M* = 3.7 compared to 4.1) [19]. Our sample appears less problematic than previously reported in the literature where adolescents with T1D were found to have more problem behavior compared to healthy peers [7–10]. Possible explanations for this discrepancy could be that we included a slightly younger group compared to the SDQ validation study, a selection bias, or the fact that previous research was conducted a decade ago. Nevertheless, almost 15% of the adolescents in our study reported levels of problem behavior above the normal range. This underscores the clinical relevance of our proposed model.

The relationship between more behavior problems and suboptimal HbA1c levels has been demonstrated in other studies as well [12–14]. The present study confirms our hypothesis that this relationship is mediated by confidence and self-management of diabetes. More behavioral problems seem to decrease the adolescent's confidence in the management of their diabetes, in concordance with previous research [17]. The need to address psychosocial issues in pediatric diabetes care is recognized [29, 30]. Psychosocial well-being is an important outcome in and of itself but also has clear relevance to understanding problems in achieving satisfactory glycemic control [31, 32]. Timely detection and management of psychosocial issues, however, have been shown to be difficult in routine care, where time is limited and measurements of adolescents' physical health often have priority [33]. Our findings corroborate the clinical relevance of finding practical ways to ensure that assessment and management of behavioral problems in adolescents are in place.

### 4.1. Limitations

Although the current study contributes to enhancing our understanding of the complex interactions between psychosocial and biological developmental trajectories, some limitations should be taken into account. First, our study was cross-sectional and we cannot infer causality. The 12-year study of Northam et al. examined the relationship between problem behavior at diagnosis and longtime poor glycemic control but did not look at possible mediating pathways [16]. Future longitudinal research is planned to examine this relationship in more detail. Moreover, the relationship between the psychosocial development and diabetes outcomes is multifaceted. In the present study we only tested the contribution of a few of the factors involved and took HbA1c as a marker for glycemic control. In addition, we may want to explore the momentary impact of behavior problems on blood glucose fluctuations which is likely to exist. Conversely, high and low blood glucose values can influence the adolescents' behavior, thereby creating a vicious cycle of events [34]. With regard to the measurements, we chose to administer the questionnaires via the internet, for pragmatic reasons. Also online administration of questionnaires is patient-friendly and more appealing to adolescents than traditional paper-and-pencil. We should acknowledge that we cannot validate that respondents have all filled in the online questionnaire without interference from others (e.g., parent, siblings); however, several studies have shown over the years that questionnaires completed via the internet are as reliable as paper-and-pencil [35, 36]. The internal consistency (Cronbach's *α*) of the adapted version of the mismanagement scale proved to be relatively low in our study (*α* = 0.47). This may be due to the fact that management behaviors are relatively independent of one another or due to the small number of items, as the adapted version of the questionnaire consists of 4 rather than the 10 items in the original questionnaire. Psychometric validation of the scale warrants further research.

## 5. Conclusion

More problem behavior in adolescents with type 1 diabetes is associated with worsened glycemic control and this relationship is mediated by low confidence in diabetes self-care and poorer self-management of the diabetes. This finding has clinical implications. Psychosocial screening should include both internal problem behavior and external problem behavior. To assist adolescents in achieving better glycemic control, it would seem imperative to help them build their confidence and reduce diabetes mismanagement, for example, improving their self-care practices.

## Figures and Tables

**Table 1:** Characteristics of participating adolescents.

Boys (N/%)	45 (51.1)
Age (yrs)	12.9 ± 1.2
HbA1c	64.3 mmol/mol (8.0%) ± 11.5 mmol/mol
Age diabetes onset	7.1 ± 3.8
Diabetes duration (yrs)	5.8 ± 3.8
Pump/injections (%)	80.7/19.3 ≥ 4 per day
Traditional family composition (%)	83
SDQ overall problem behavior (0–40)	8.6 ± 4.3
SDQ external problem behavior (0–20)	4.9 ± 2.8
SDQ internal problem behavior (0–20)	3.7 ± 2.8
CIDS-Youth (12–60)	51.2 ± 5.3
Mismanagement (4–16)	6.4 ± 2.0

Data are means ± SD, unless otherwise indicated.
